# Selecting Better Biocatalysts by Complementing Recoded Bacteria**

**DOI:** 10.1002/anie.202213942

**Published:** 2022-12-07

**Authors:** Rudy Rubini, Suzanne C. Jansen, Houdijn Beekhuis, Henriëtte J. Rozeboom, Clemens Mayer

**Affiliations:** ^1^ Stratingh Institute for Chemistry University of Groningen Nijenborgh 4 9747 AG Groningen The Netherlands; ^2^ Biomolecular Sciences and Biotechnology Institute University of Groningen Nijenborgh 4 9747 AG Groningen The Netherlands

**Keywords:** Carbamoylases, Directed Evolution, Genetic-Code Expansion, Synthetic Biology, In Vivo Selection

## Abstract

In vivo selections are powerful tools for the directed evolution of enzymes. However, the need to link enzymatic activity to cellular survival makes selections for enzymes that do not fulfill a metabolic function challenging. Here, we present an in vivo selection strategy that leverages recoded organisms addicted to non‐canonical amino acids (ncAAs) to evolve biocatalysts that can provide these building blocks from synthetic precursors. We exemplify our platform by engineering carbamoylases that display catalytic efficiencies more than five orders of magnitude higher than those observed for the wild‐type enzyme for ncAA‐precursors. As growth rates of bacteria under selective conditions correlate with enzymatic activities, we were able to elicit improved variants from populations by performing serial passaging. By requiring minimal human intervention and no specialized equipment, we surmise that our strategy will become a versatile tool for the in vivo directed evolution of diverse biocatalysts.

## Graphical abstract

An in vivo selection strategy is presented, in which bacteria addicted to non‐canonical amino acids (ncAAs) are complemented by enzymes that can yield these building blocks from synthetic precursors. As growth rates under selective conditions correlate with enzyme activities, serial passaging elicited better biocatalysts from populations harboring enzyme libraries. The platform was used to improve the activity of carbamoylases for ncAA‐precursors.

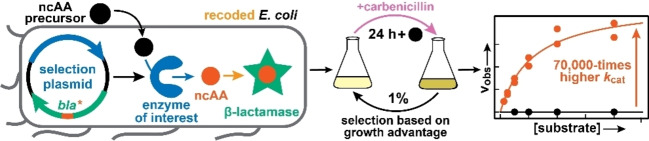

## Introduction

**Figure 1:**
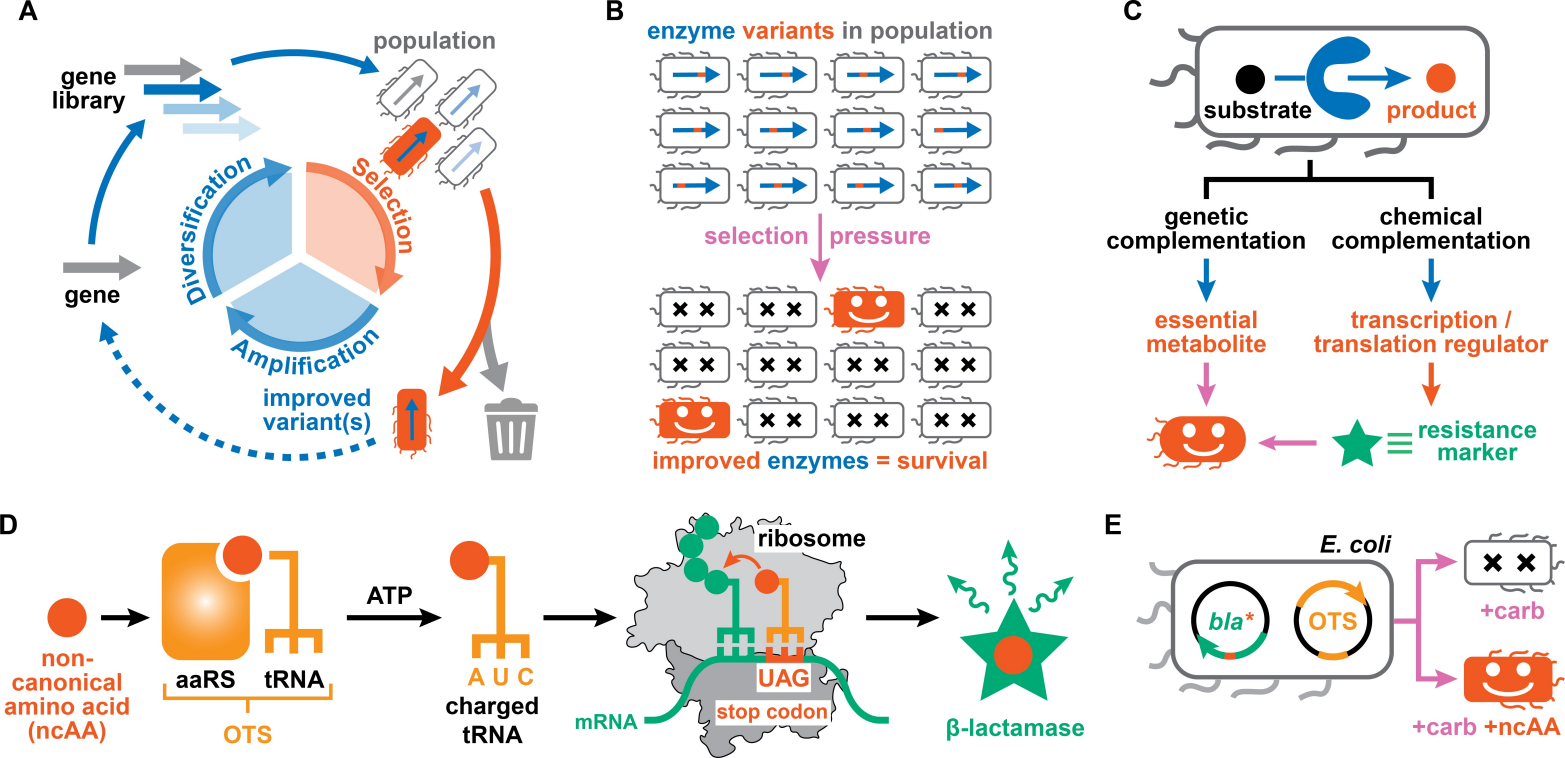
Selection strategies in the directed evolution of biocatalysts. A) The properties of biocatalysts are tailored in directed evolution via iterative cycles of gene diversification, selection, and amplification. B) In selections, enzyme variants in populations are assessed all at once with an applied selection pressure eliminating all undesired library members. C) Selections make use of genetic or chemical complementation strategies to link the activity of an enzyme of interest to the survival/growth of a producing organism. D–E) Orthogonal translation systems (OTSs) consist of aminoacyl tRNA synthetase (aaRS)/suppressor tRNA pairs that facilitate the site‐selective incorporation of non‐canonical amino acids into proteins of interest. When engineering OTSs for ncAAs, functional aaRS/tRNA pairs are identified by allowing *E. coli* to survive in presence of β‐lactam antibiotics (e.g. carbenicillin, carb). Panel D was adapted from Ref. [17].

Enzymes are marvelous catalysts that accelerate reactions with unmatched rates and selectivities.^[1]^ Their use as standalone catalysts or integration into designer microbes promises the sustainable production of fine chemicals, biofuels, and biomedicines.^[2]^ However, enzymes found in nature are rarely optimal for industrial applications with low stabilities, narrow reaction scopes and/or low activities on industrially‐relevant substrates restricting their applicability. Courtesy of advances in molecular and structural biology, it has become feasible to tailor enzyme properties by mimicking the Darwinian algorithm in the laboratory (Figure 1A).^[3]^ One round of the *directed evolution* of biocatalysts comprises three steps: (1) creating diversity by introducing mutations, (2) identifying enzyme variants with improved characteristics and (3) amplifying selected variants. When performed iteratively, these steps enable the gradual improvement of an enzyme's catalytic performance.^[4]^ Applying this algorithm for enzyme engineering has proven useful for increasing stability, identifying specialized biocatalysts for non‐natural substrates, boosting promiscuous or new‐to‐nature activities, and altering the stereo‐ or regioselectivities of enzymes.

Over the past decades, mutagenesis, recombination, and computational tools have sufficiently matured to enable the generation of large gene libraries with billions of variants.^[5]^ However, navigating the resulting sequence space efficiently remains critically dependent on identifying improved variants based on the (high‐throughput) analysis of target products.^[6]^ Fundamentally, the strategies employed to analyze gene libraries can be divided into either screens or selections.^[4]^ Screens necessitate the individual assessment of every library member, with throughput thus being limited by the speed of the employed biochemical or biophysical readout.^[7]^ In contrast, selections link the function of an enzyme of interest to the survival or growth of a host organism.^[8]^ Large populations of up to 10^9^ individual cells can be assessed all at once, as an applied selection pressure eliminates all of the undesired library members (Figure 1B).^[9]^ Exemplified by the ease with which antibiotic resistance genes can be evolved in the laboratory,^[10]^ selections remove much of the time, cost and technical challenges associated with assaying vast libraries. However, transformations of interest for biotechnological applications rarely provide a growth advantage to a producing organism.

Successful in vivo selections thus rely on linking the host organism's fitness to the genotype of an enzyme of interest, with the cell acting as both segregated compartment and expression host.[^8^, ^9^] Such a genotype‐phenotype link can be achieved either via *genetic* or *chemical complementation* (Figure 1C). In genetic complementation, the inability of the host organism to synthesize an essential metabolite (auxotrophy) is exploited to identify protein catalysts that are able to yield this compound. As a result, these efforts are restricted to a small set of enzymes for which an auxotrophic host is available or can be constructed by deleterious mutagenesis.^[11]^ In chemical complementation, an enzyme's activity is linked to the production of a genetically‐encoded reporter that enables cellular survival (Figure 1C).^[12]^ By often relying on antibiotic resistance markers as reporter genes, this type of complementation avoids the use of auxotrophic strains and can theoretically be applied to a wider repertoire of enzymatic reactions. Traditionally, such selections function by small‐molecule products of an enzymatic transformation binding either to a transcription factor or a genetically‐encoded riboswitch, thereby regulating the transcription or translation of the employed reporter gene.^[13]^ More recently, linking enzymatic activities to phage propagation has resulted in a means to evolve enzymes autonomously along extended evolutionary trajectories.^[14]^

Additionally, a form of chemical complementation is employed when engineering orthogonal translation systems (OTSs) with the ability to expand the genetic code of organisms (Figure 1D).^[15]^ Specifically, the activity of aminoacyl tRNA synthetase (aaRS) variants to load suppressor tRNAs with non‐canonical amino acids (ncAAs) is routinely assessed by the suppression of an in‐frame stop codon within an antibiotic resistance gene, such as a β‐lactamase.^[16]^ Improved aaRS/tRNA pairs are therefore readily identified as they allow *Escherichia coli* to grow under higher concentrations of a β‐lactam antibiotic (Figure 1E). Notably, we recently demonstrated that this link between the activity of an OTS and host survival in presence of ampicillin can serve as a readout of unrelated catalytic activities.^[17]^ Specifically, we constructed a biocontainment strategy, in which new‐to‐nature reactions gated bacterial growth by transition‐metal complexes that promote the formation of ncAAs.

In analogy, we surmised that the ncAA‐dependency of recoded organisms could also serve as a starting point for engineering enzymes that can yield these essential building blocks from appropriate precursors. Here we provide proof‐of‐concept for such a chemical complementation strategy by demonstrating the in vivo directed evolution of carbamoylases, an enzyme class relevant for the biotechnological production of enantiopure (un)natural amino acids.^[18]^ In our directed evolution platform, the growth rates of *E. coli* correlated with the activity of carbamoylase variants they produce. Our strategy enabled us to identify enzymes that display turnover frequencies and catalytic proficiencies more than five orders of magnitude higher than those observed for the wild‐type carbamoylase. Lastly, we also demonstrate that differences in growth rates provide a means to select improved variants from populations by performing serial passages under selective conditions. Given the ease with which improved carbamoylases were identified within our platform, we surmise that the use of recoded organisms in chemical complementation systems should facilitate the in vivo and continuous directed evolution of diverse biocatalysts.

## Results and Discussion

### Design of the Complementation System

**Figure 2:**
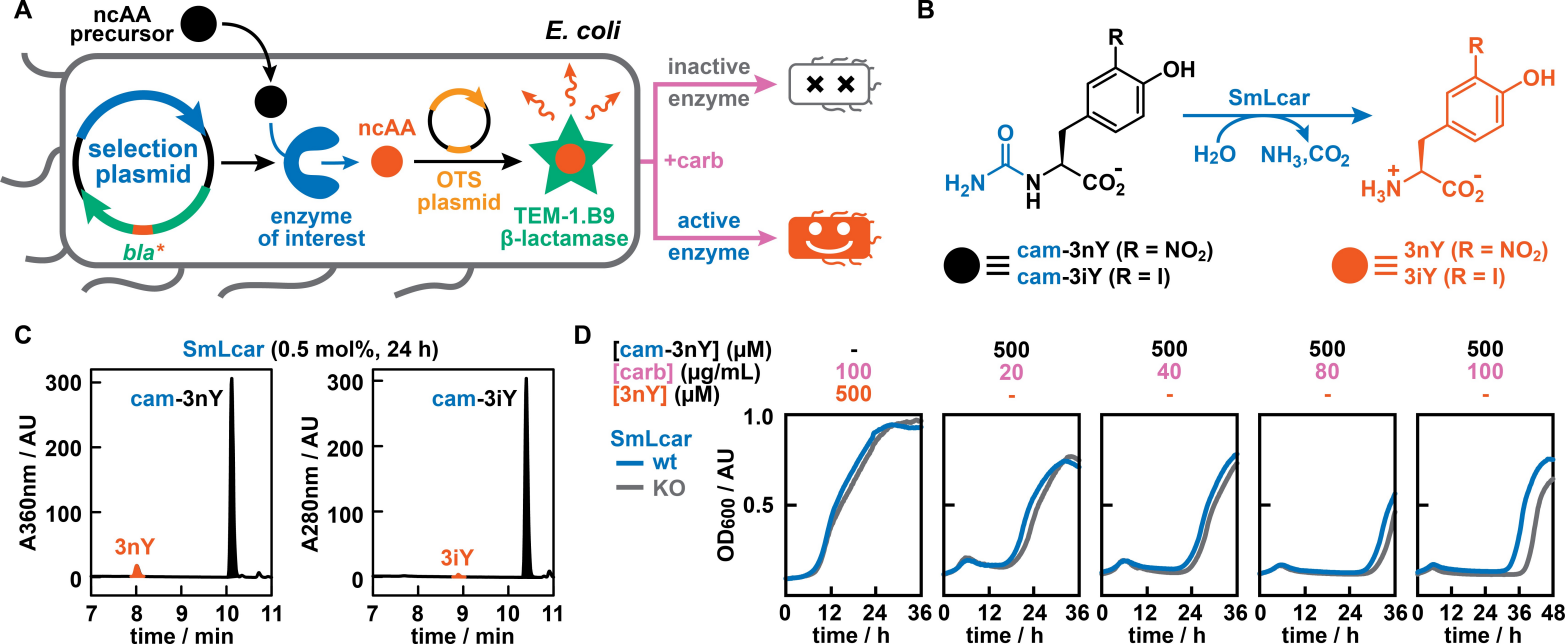
Design and validation of an in vivo enzyme engineering platform. A) Blueprint of a chemical complementation system based on recoded bacteria. A precursor is converted by an enzyme of interest to yield a ncAA, which is incorporated into a β‐lactamase. Active enzymes should allow *E. coli* to grow in presence of carbenicillin, while inactive ones perish under the selection pressure. B) Model transformations catalyzed by SmLcar that give rise to the ncAAs *l*‐3‐nitro‐tyrosine (3nY) and *l*‐3‐iodo‐tyrosine (3iY). C) HPLC chromatograms showing SmLcar is a weakly active catalyst for the conversion of carbamoylated ncAAs. D) Growth curves of *E. coli* in presence of varying concentrations of carbenicillin and 3nY or cam‐3nY. The weak activity of SmLcar for the ncAA precursor provides a small growth advantage in the complementation system, when compared to an inactive enzyme variant (SmLcar_KO).

Our directed evolution platform is based on the complementation of ncAA‐dependent *E. coli* cells with enzymes that yield these essential compounds from appropriate precursor molecules (Figure 2A). Establishing this functional link between enzymatic activity and survival requires the introduction of three genetic components: (1) an enzyme able to convert an appropriate precursor to the ncAA (=input); (2) an OTS selective for this ncAA (=sensor); and (3) a β‐lactamase that can degrade carbenicillin (=readout). To avoid false positives that can result from the spontaneous mutation of the in‐frame stop codon within the resistance gene, we make use of the engineered β‐lactamase variant, TEM‐1.B9,^[19]^ whose activity to degrade carbenicillin is strictly dependent on the incorporation of certain ncAAs, such as *l*‐3‐iodo‐ or *l*‐3‐nitro‐tyrosine (3iY or 3nY). Following the production of all three components in *E. coli*, varying the concentration of carbenicillin should provide a tunable *selection pressure*, as more active enzymes should be able to proliferate in presence of higher antibiotic concentrations.

As a model biocatalyst class, we selected carbamoylases, which promote the hydrolytic cleavage of *N*‐carbamoyl‐amino acids (Figure 2B).^[18]^ Together with hydantoinases, they make up a scalable and widely‐employed enzymatic cascade for the production of enantiopure (un)natural amino acids (=hydantoin process).^[20]^ Based on its reportedly moderate activity for *N*‐carbamoylated‐*l*‐tyrosine, we produced and purified SmLcar, a carbamoylase from *Sinorhizobium meliloti* strain CECT 4114.^[21]^ When challenged with the synthetic ncAA precursor *N*‐carbamoyl‐*l*‐3nY (cam‐3nY, 2 mM), SmLcar (10 μM) yielded 6.1 % of the ncAA after 24 hours at pH 6 (Figure 2C). Conversely, *N*‐carbamoyl‐*l*‐3iY (cam‐3iY) proved a more challenging substrate, with SmLcar failing to yield detectable levels of 3iY at pH 6 and only providing 0.8 % of the ncAA at pH 8 under otherwise identical conditions.

A weakly active biocatalyst in hand, we assembled our chemical complementation system by introducing a selection and an OTS plasmid in *E. coli* (Figure 2A). Both TEM‐1.B9 and SmLcar were placed on a modified pACYCDuet‐1 backbone (pACYC_GG), which features two type IIS restriction sites for the modular exchange of the target enzyme and/or the β‐lactamase (see Supporting Information for details). The OTS—here consisting of the 3iY‐aaRS and a suppressor tRNA—is encoded on the established pULTRA plasmid system, which features a resistance marker and an origin of replication that are compatible with the second vector.^[22]^ Lastly, the expression of all three genetic components is dependent on the addition of isopropyl‐β‐d‐1‐thiogalactopyranoside (IPTG), thus enabling the timed transcription and/or translation of all elements in our chemical complementation system.

### Validation of the Complementation Strategy

Before attempting to boost the activity of SmLcar for the synthetic ncAA precursor cam‐3nY, we aimed to verify whether bacterial survival in presence of carbenicillin was dependent on (1) the supply of 3nY or (2) the addition of cam‐3nY in combination with the production of SmLcar. In these experiments, we monitored bacterial growth at 30 °C by following the optical density at 600 nm (OD_600_) over a period of up to 48 hours (see Supporting Information for details). Performing these experiments in 96‐well plates enabled us to record the growth curves of cultures under varying conditions in parallel. To pinpoint the differences between an active and inactive biocatalyst, we also prepared the knock‐out variant SmLcar_KO, in which arginine 292, an essential residue for substrate recognition, is replaced with an alanine.^[21b]^

As expected, *E. coli* cultures featuring the OTS and TEM‐1.B9 displayed robust growth upon addition of 3nY (500 μM) and carbenicillin (up to 500 μg mL^−1^) to the media (Figure S1 in the Supporting Information). The observed lag times were independent of the antibiotic concentration and comparable to those observed for cultures lacking carbenicillin (≈5–8 hours). Conversely, the addition of cam‐3nY (500 μM) to SmLcar or SmLcar_KO containing bacteria resulted in extended lag phases, whose lengths increased with higher carbenicillin concentrations (Figure 2D). As we do not observe growth in absence of cam‐3nY (Figure S2) at carbenicillin concentrations exceeding 20 μg mL^−1^, we ascribe the observed proliferation of *E. coli* harboring SmLcar_KO to the hydrolysis of cam‐3nY that is promoted by endogenous enzymes with weak promiscuous activities for this synthetic substrate. Notably though, cells producing SmLcar consistently displayed somewhat shorter lag times than those featuring the knock‐out variant, a trend that became more pronounced at higher carbenicillin concentrations (Figure 2D). While the growth advantage provided by the active carbamoylase is small, we anticipated that the production of SmLcar variants featuring beneficial mutations should result in *E. coli* strains displaying significantly shorter lag times.

### Directed Evolution of SmLcar

**Figure 3:**
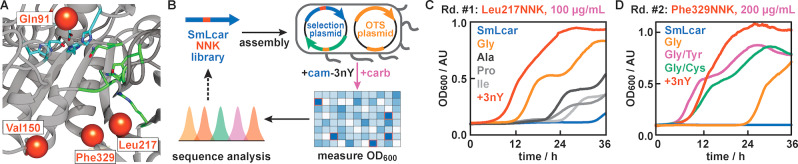
Directed evolution of SmLcar. A) Close‐up view of the active site of SmLcar. Red spheres indicate positions which were targeted by site‐directed mutagenesis in the evolution. Residues critical for binding of the two bivalent metal ions are highlighted in blue and amino acids that are either conserved or involved in substrate binding are shown in green. B) Workflow for the directed evolution of SmLcar in 96‐well plates. C) Growth curves of enzyme variants with substitutions at Leu217 that provided a growth advantage in the first round of evolution. D) Growth curves of improved SmLcar variants featuring an additional mutation at Phe329. Note that increased carbenicillin concentrations result in longer lag times for SmLcar_G and the wild‐type being unable to proliferate. Entries specified as +3nY refer to a positive control where cells producing SmLcar grow in presence of the ncAA (500 μM) instead of its synthetic precursor.

To identify positions in SmLcar that can be targeted by mutagenesis to boost the activity for cam‐3nY, we predicted the structure of SmLcar using *AlphaFold2* (Figure 3A, see Supporting Information for details).^[23]^ Based on the available structural and functional information for carbamoylases,^[18]^ we first identified residues presumed to coordinate the pair of bivalent metal ions (e.g. Zn^2+^, Ni^2+^, or Mn^2+^) and those critical for substrate binding.

From there, we selected four positions, which are either in proximity to the catalytic site (Gln91) or line the predicted binding pocket (Val150, Leu217, and Phe329, Figure 3A). Libraries targeting each position were constructed using primers featuring degenerate NNK codons and 84 variants per residue were evaluated in parallel by following the OD_600_ in a plate reader in presence of 500 μM cam‐3nY and 100 μg mL^−1^ carbenicillin (Figure 3B, see Supporting Information for details).

Gratifyingly, for the library targeting position Leu217, we detected several *complementation events*, as judged by the presence of significantly higher cell densities after 27 hours than those observed for the wild‐type protein (Figure S3). Sequencing the SmLcar gene on their selection plasmids revealed that these variants featured common amino acid substitutions (Gly, Ala, Pro, or Ile), with the three variants displaying the highest OD_600_ values all being Leu217Gly. Inspecting the growth curves of individual variants over 36 hours further attests that all complementing clones displayed lag times that are shorter than those observed for the wild‐type enzyme (Figure 3C and Figure S3), with the glycine variant, SmLcar_G, again proving to be the fittest (lag time of ≈12 hours). Curiously, we observed a biphasic growth curve upon complementation for all variants. We ascribe this phenomenon to the production of SmLcar variants being toxic to the cells, as upon lowering the inducer concentration, only a single exponential phase could be observed (see Supporting Discussion for details).

Next, we selected SmLcar_G as a template for an additional round of site‐directed mutagenesis, in which we targeted the remaining three positions for randomization (Figure 3A). While the library of Gln91 did not result in a fitness increase, randomization of both Val150 and Phe329 yielded a number of hits that displayed faster growth than SmLcar_G (Figure S4). We selected the best‐performing clones for each position and subjected them in triplicates to a rescreen under higher carbenicillin concentration (200 μg mL^−1^, Figure S5). Consistent with a more stringent selection pressure, lag times for SmLcar_G increased to ≈24 hours, while the wild‐type enzyme was unable proliferate under these conditions. Conversely, the rescreen identified two variants featuring either a Phe329Tyr or Phe329Cys substitution with lag times of only ≈8 hours (Figure 3D and Figure S5). In fact, *E. coli* producing the engineered carbamoylases SmLcar_GY or SmLcar_GC displayed growth rates and lag times that are comparable to those observed upon direct supplementation of 3nY to recoded bacteria.

### Characterization of Improved SmLcar Variants

**Figure 4:**
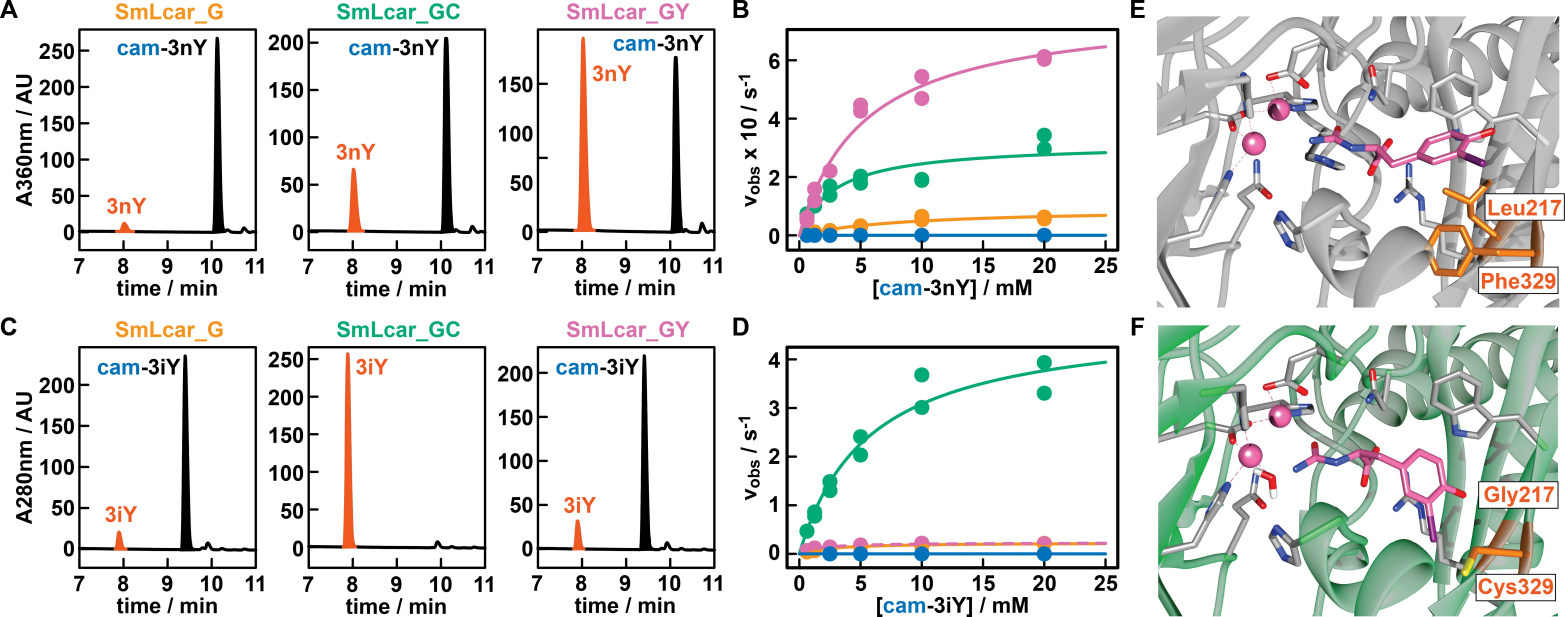
Characterization of improved SmLcar variants. A) HPLC chromatograms showing the activities of engineered SmLcar variants for cam‐3nY. B) Saturation kinetics for SmLcar (blue), SmLcar_G (orange) and SmLcar_GY (purple) for cam‐3nY. The corresponding kinetic parameters obtained from fitting data points to the Michaelis Menten equation are listed in Table 1. C) HPLC chromatograms showing the activities of engineered SmLcar variants for cam‐3iY. D) Saturation kinetics for SmLcar (blue), SmLcar_G (orange), SmLcar_GY (purple), and SmLcar_GC (green) for cam‐3iY. The corresponding kinetic parameters obtained from fitting data points to the Michaelis Menten equation are listed in Table 1. E–F) Close‐up view of the active sites of SmLcar (E, PDB: 8APZ) and SmLcar_GC (F, PDB: 8AQ0) with cam‐3iY docked into the obtained crystal structures.

To demonstrate that shorter lag times of *E. coli* correlate with higher activities of SmLcar variants identified in the screen, we produced and purified these carbamoylases and evaluated their performance on ncAA precursors in vitro. While the wild‐type enzyme at 10 μM (0.5 mol%) yielded a modest 6.1 % of the ncAA from cam‐3nY (500 μM) over 24 hours (Figure 2C), all variants identified in the evolution campaign were able to fully convert the synthetic precursor under these conditions. Shortening the reaction time to 1 hour and lowering the enzyme concentration 20‐fold to 0.5 μM revealed that lag‐times in 96‐well plates accurately report on the activity of engineered carbamoylase variants. Specifically, SmLcar_G, the best performing variant in the first round, and the double mutants SmLcar_GC and SmLcar_GY gave 3nY in 4.5 %, 38.8 % and 50.5 % yield, respectively (Figure 4A).

Next, we determined the kinetic parameters of SmLcar and the best variants from each round to accurately pinpoint their level of improvement (Figure 4B and Table 1). As expected, the parent enzyme displayed a low turnover frequency (*k*_cat_=1.05×10^−4^±1.01×10^−5^ s^−1^) and catalytic proficiency (*k*_cat_/*K*_M_=3.54±0.98×10^−2^ M^−1^ s^−1^). Notably, the single Leu219Gly substitution in SmLcar_G provided a >850‐fold boost in turnover frequencies (*k*_cat_=0.093±0.014 s^−1^) and resulted in a ≈300‐fold increase in catalytic proficiencies (*k*_cat_/*K*_M_=10.4±2.6 M^−1^ s^−1^). The addition of the Phe329Tyr substitution in SmLcar_GY also proved beneficial, providing a further 8.4‐ and 14.2‐fold increase in *k*_cat_ (=0.78±0.05 s^−1^) and *k*_cat_/*K*_M_ (=148±12.9 M^−1^ s^−1^), respectively.

**Table 1:** Kinetic parameters of SmLcar and engineered variants for the ncAA precursors cam‐3nY and cam‐3iY.

enzyme	cam‐3‐*l*‐nitrotyrosine (cam‐3nY)				cam‐3‐*l*‐iodotyrosine (cam‐3iY)			
	*k*_cat_ [s^−1^]	*K*_M_ [mM]	*k*_cat_/*K*_m_ [M^−1^ s^−1^]	relative	*k*_cat_ [s^−1^]	*K*_M_ [mM]	*k*_cat_/*K*_M_ [M^−1^ s^−1^]	relative
SmLcar	1.05×10^−4^ (1.01×10^−5^)	2.98 (0.87)	3.54×10^−2^ (9.77×10^−3^)	1	6.92×10^−5^ (2.09×10^−5^)	13.8 (7.91)	5.00×10^−3^ (3.15×10^−3^)	1
SmLcar_G	9.29×10^−2^ (1.36×10^−2^)	8.91 (2.81)	10.4 (2.58)	294	0.232 (0.013)	2.58 (0.46)	89.9 (13.2)	1.80×10^4^
SmLcar_GY	0.78 (0.05)	5.30 (0.88)	148 (12.9)	4 170	0.231 (0.05)	1.45 (0.16)	159 (14.2)	3.18×10^4^
SmLcar_GC	0.32 (0.034)	2.92 (0.95)	108 (34.8)	3 060	4.84 (0.39)	5.71 (1.14)	849 (127)	1.70×10^5^

Curious whether the substitutions identified in the directed evolution present a more general solution for the conversion of *m*‐substituted tyrosine analogs, we determined the activity of engineered carbamoylases for cam‐3iY. Indeed, SmLcar_G (10 μM) was able to fully convert the synthetic precursor (500 μM) within 24 hours at pH 8, while the wild‐type enzyme gave only 0.8 % yield under identical conditions. To obtain a representative comparison between our evolved carbamoylases, we again lowered the enzyme concentration to 0.5 μM and shortened reaction times to 1 hour (Figure 4C). Surprisingly, SmLcar_G and SmLcar_GY gave rise to comparable yields (7.3 % and 8.0 %, respectively), indicating that the improved activity of the tyrosine variant for cam‐3nY reflects a certain specialization toward the screening substrate. Unexpectedly, the Phe329Cys substitution resulted in significantly improved reactivity, as SmLcar_GC fully converted cam‐3iY under these conditions (Figure 4C).

Determining the steady‐state kinetic parameters confirmed the increased activity of all variants for cam‐3iY, when compared to the wild‐type (*k*_cat_=6.92×10^−5^±2.09×10^−5^ s^−1^; *k*_cat_/*K*_M_=5.00×10^−3^±3.15×10^−3^ M^−1^ s^−1^). The Leu217Gly substitution again proved crucial and boosted the turnover frequency (=0.23±0.01 s^−1^) and catalytic efficiency (89.9±13.2 M^−1^ s^−1^) by more than three and four orders of magnitude, respectively (Figure 4D and Table 1). Consistent with the conversions obtained by HPLC, the addition of Phe329Tyr resulted only in a minor improvement in catalytic proficiency (=159±14.2 M^−1^ s^−1^), when compared to SmLcar_G. In stark contrast, installing a cysteine at position 329 proved highly beneficial, boosting the turnover number (4.84±0.39 s^−1^) and the catalytic efficiency (849±127 M^−1^ s^−1^) by another order of magnitude. When compared to the parent carbamoylase, SmLcar_GC displays a ≈70 000 times higher *k*_cat_ and a ≈170 000‐fold improvement of *k*_cat_/*K*_M_. Combined, these results attest that our strategy of linking unrelated enzymatic activities to bacterial proliferation presents an effective means to engineer weakly active biocatalysts that do not fulfill a metabolic role.

### Crystal Structures of SmLcar and SmLcar_GC

To rationalize the massive performance improvements of our engineered carbamoylases, we obtained crystal structures for SmLcar and the double mutant SmLcar_GC (Figure S6 and see Supporting Information for details). Guided by the electron densities of unknown small molecules bound to the active sites of SmLcar and SmLcar_GC (Figure S7), we performed docking studies (*YASARA*)^[24]^ with the synthetic precursor, cam‐3iY, for both enzymes. Comparing the resulting structures of the wild‐type and the evolved variant revealed a striking difference in the orientation of the substrate in their respective binding pockets (Figures 4E–F). Specifically, the critical Leu217Gly substitution identified in the first round enlarges the binding pocket and allows the aromatic side chain to flip by ≈90°. While our kinetic characterization reveals that this does not drastically alter the affinity of the ncAA precursor for the enzyme, as judged by the small differences in *K*_M_, this conformation appears to be highly beneficial for the attack of water at the adjacent metal‐binding site. Lastly, position 329 is distal to Gly217 and the introduced cysteine in SmLcar_GC (or Tyr in SmLcar_GY) interacts with the substrate to likely further lock it in an active conformation.

### Recapitulating the Evolution of SmLcar_GY on the Population Level

In developing a robust, chemical complementation strategy we aimed to facilitate the *selection* of improved biocatalysts from diverse populations. Overcoming the need of assessing the activity of enzyme variants one by one is highly desirable, as selections combine a high throughput with operational simplicity.[^8^, ^9^] In our platform, the fitness of *E. coli* in presence of carbenicillin and cam‐3nY is dictated by the activity of the produced carbamoylase variant. With improved biocatalysts resulting in shorter lag times under these selective conditions, *E. coli* harboring such variants should outcompete bacteria featuring less active carbamoylases. Relying on this robust genotype‐phenotype link, we set out to provide proof‐of‐concept that our platform is amenable to elicit improved enzymes from populations based on the growth advantage they bestow on a producing organism.

**Figure 5:**
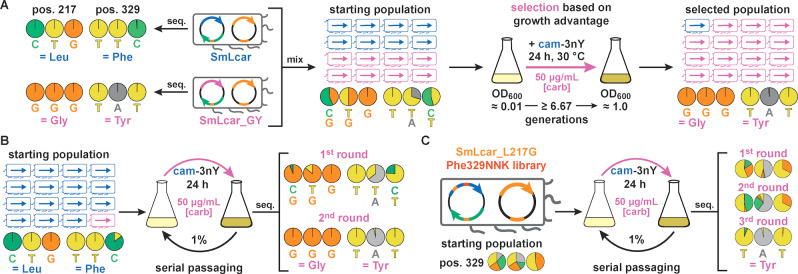
Selection of improved carbamoylases on the population level. A) Mock selection containing an equal mixture of SmLcar and SmLcar_GY. Overnight growth under selective conditions results in the improved carbamoylase becoming the dominant species. Base calls from Sanger sequencing of individual variants or populations are displayed as pie charts. B) Mock selections for a ≥10 : 1 mixture of SmLcar and SmLcar_GY. Cultures grown overnight are subjected to serial passaging under selective pressure. Two such growth‐dilution cycles are sufficient to yield a population that is dominated by SmLcar_GY. C) Application of the selection scheme by serial passaging to the second‐round SmLcar_G_Phe329NNK library. Starting from a diverse library, three growth‐dilution cycles elicit SmLcar_GY as the dominant variant as judged by Sanger sequencing.

Toward this end, we first performed a mock selection by mixing cultures containing the wild‐type carbamoylase and SmLcar_GY in a 1 : 1 ratio (Figure 5A and Table S1 in the Supporting Information). Isolating the selection plasmid and analyzing the carbamoylase gene encoded on it by Sanger sequencing allows us to (1) unambiguously assign either variant, (2) estimate their relative frequency within a given a population, and (3) track changes in the population over time. Prior to selection, the mixed population (OD_600_≈0.01) reflected the anticipated 1 : 1 ratio between the wild‐type and SmLcar_GY, which was subsequently challenged to grow overnight in presence of cam‐3nY (500 μM) and carbenicillin (50 μg mL^−1^). Sequencing the plasmids isolated from the densely‐grown culture the next day revealed that the population had become dominated by SmLcar_GY (Figure 5A). As such, this result attests that the growth advantage provided by a more active carbamoylase under selection conditions enables its amplification in a mixed population.

To test the ability of our platform to identify better biocatalysts that are less abundant, we performed another mock selection, in which wild‐type and SmLcar_GY cultures were mixed in a ≥10 : 1 ratio (Figure 5B). Sequencing the starting population failed to detect appreciable levels of the improved carbamoylase, a fact that likely reflects the limitations of Sanger sequencing. This heavily‐skewed population was subjected to *serial passaging*, that is cultures were diluted by a factor of 100 following growth under selective conditions for 24 hours. Again, we traced the fate of the population by sequencing plasmids recovered after each growth period. Strikingly, within 24 hours SmLcar_GY became the dominant variant in the population, with an additional dilution‐growth step resulting in a population devoid of the wild‐type carbamoylase (Figure 5B and Table S1). To exclude the possibility of SmLcar_GY being amplified independent of its catalytic activity, we also performed serial dilutions on the mixed population by adding 3nY to the growth media. As expected, in absence of selecting for catalytic activity, sequencing cultures following two dilution‐growth cycles did not show a significant change in the population (Figure S8).

Combined, the amplification factors observed for SmLcar_GY in the mock selections attest on a robust genotype‐phenotype link that allows individual cells rather than the whole population to benefit from producing a more proficient enzyme. Consequently, *E. coli* cells harboring less active carbamoylases cannot strongly exploit escape mechanisms, such as beginning to divide once carbenicillin has been (largely) degraded in the media or taking up 3nY that is released by fitter bacteria. Avoiding these unintended amplification and escape mechanisms is critical for the successful adaptation of our platform to select improved carbamoylases from libraries.

Encouraged by these observations, we challenged our platform to elicit SmLcar_GY from the SmLcar_G_Phe329NNK library we used in the second round of evolution in 96‐well plates (Figure 5C). Unlike in mock selections, where we employ the far inferior wild‐type carbamoylases, the activity differences in this population are significantly smaller, with SmLcar_G and SmLcar_GC being about 10 % and 50 % as active as the desired tyrosine variant (cf. Figure 4A and Table 1). Sequencing the starting population attested on an equal distribution of all desired nucleotides for the NNK stretch that encodes for all 20 amino acids at position 329. As before, we performed serial passages under selective and non‐selective conditions and followed changes in the population by sequencing. As expected, the culture grown in presence of 3nY did not undergo significant changes following two growth‐dilution cycles (Figure S9). Conversely, the population subjected to selective conditions was dynamic, with the codon TAT corresponding to tyrosine ultimately dominating the population following three passages (Figure 5C and Table S1). To independently verify this result, we sequenced ten individual colonies from the population and found that six of them indeed encoded for SmLcar_GY (Table S2). Lastly, applying the same selection mechanism to the first‐round library SmLcar_Leu217NNK also elicited SmLcar_G as the dominant variant following three growth‐dilution cycles (Figure S9).

## Conclusion

Utilizing in vivo selections for the directed evolution of biocatalysts promises to quantitatively capture enzyme activity and efficiently isolate desired variants from diverse populations.[^8^, ^9^] Adopting powerful in vivo selections for enzymes that do not fulfill a metabolic function requires the introduction of genetically‐encodable elements that link cellular survival to a transformation of interest.^[13b]^ Here, we demonstrate that ncAA‐dependent organisms represent an ideal platform for constructing such chemical complementation systems that can be employed for enzyme engineering purposes. Specifically, we showcase that bacterial proliferation is dependent on an enzyme that can provide the ncAA from an appropriate precursor and that the growth advantage provided by variants is dependent on their activities.

Using this artificial link between enzyme activity and bacterial proliferation, we evolved the carbamoylase SmLcar to accept carbamoylated *m*‐substituted tyrosine analogs. Using bacterial growth in presence of carbenicillin as the *only* readout allowed us to identify SmLcar variants that hydrolyzed ncAA precursors with catalytic proficiencies up to five orders of magnitude higher than those observed for the parent enzyme. While the initial evolution was performed in 96‐well plates, the fact that our platform does not require any handling steps following inoculation significantly simplifies and streamlines the discovery of carbamoylases with improved kinetic parameters. Furthermore, we demonstrated that the growth advantage provided by improved carbamoylases is largely confined to producing cells and does not increase the fitness of less active variants in a population. This robust genotype‐phenotype link enabled us to elicit improved carbamoylases in mock selections and recapitulate the evolution of SmLcar on the population level. These selections were performed by straightforward serial passaging following overnight growth, thus further minimizing the handling steps for the experimentalist. Moreover, the fact that our platform is not dependent on specialized and/or expensive equipment argues well for its widespread application.

In the future, we will explore the full capabilities of our chemical complementation system, which promises the following developments. First, building on well‐established strategies to expand the genetic code of *E. coli*, our platform is applicable to any reaction that can be linked to the formation of one of the >150 non‐canonical building blocks that have been incorporated into proteins in vivo.^[16]^ Critically, by having the potential to provide the same readout (=survival) for reactions catalyzed by mechanistically‐diverse enzymes, our platform is not only innovative but also flexible in nature. Second, taking advantage of the operational simplicity and realizing the high throughput of biological selections will allow for a thorough interrogation of the available sequence space. As such, selecting better enzymes from large and diverse libraries should facilitate the identification of beneficial mutations that cannot be rationalized or exceedingly rare mutations that work in synergy.^[4]^ Lastly, the combination of our approach with means to introduce mutations in genes of interest in vivo,[^14b^, ^25^] should allow for establishing continuous evolution approaches. By tailoring diverse enzymatic activities with minimal or without human intervention, such developments will allow us to explore long evolutionary trajectories in parallel.

## Conflict of interest

The authors declare no conflict of interest.

1

## Supporting information

As a service to our authors and readers, this journal provides supporting information supplied by the authors. Such materials are peer reviewed and may be re‐organized for online delivery, but are not copy‐edited or typeset. Technical support issues arising from supporting information (other than missing files) should be addressed to the authors.

Supporting Information

## Data Availability

The data that support the findings of this study are available from the corresponding author upon reasonable request.
